# The N-of-1 Trials and where to use them in Rheumatology

**DOI:** 10.31138/mjr.200125.ehr

**Published:** 2025-07-10

**Authors:** Maria G. Grammatikopoulou, Arriana Gkouvi, Sotirios G. Tsiogkas, Theodora Simopoulou, Dimitrios G. Goulis, Dimitrios P. Bogdanos

**Affiliations:** 1 Department of Rheumatology and Clinical Immunology, Faculty of Medicine, School of Health Sciences, University of Thessaly, Biopolis, Larissa, Greece;; 2 Unit of Reproductive Endocrinology, 1^st^ Department of Obstetrics and Gynaecology, Medical School, Aristotle University of Thessaloniki, Thessaloniki, Greece

**Keywords:** *n*-of-1, individualised care, randomised controlled trial, fibromyalgia, osteoarthritis, rheumatoid arthritis, pain, fatigue, evidence-based medicine, individualised medicine

## Abstract

**Objective:**

N-of-1 trials constitute single-patient, randomised, crossover and often, double-blind clinical trials, where each patient serves as his/her own control. The implementation of *n*-of-1 trials propels us towards the practice of patient-centric medicine, while exhibiting multiple additional advantages for rheumatology, including the identification of the most appropriate treatment for each patient, improved response, outcomes and quality of life, fewer adverse events, and reduced economic costs. The design employs similar aspects to randomised clinical trials in order to maintain scientific rigor, while producing clinically relevant treatment outcomes, tailored to each patient.

**Methods:**

For the purpose of this review, we searched PubMed and clinicaltrials.gov for *n*-of-1 trials or series conducted on patients with rheumatic diseases until August 2024.

**Results:**

N-of-1 trials can facilitate clinical decisions and evaluate the efficacy of medications, lifestyle interventions, or adjuvant treatments (i.e. for pain), while focusing on disease-specific outcomes or comorbidities (cachexia, obesity, etc.). In this review, the advantages and limitations of *n*-of-1 trials in rheumatology are discussed and trials performed on patients with rheumatic diseases are presented.

**Conclusions:**

Employing the *n*-of-1 design in everyday clinical practice consists of the epitome of patient-centred medicine, greatly benefiting patients and clinicians, facilitating deprescribing, and reducing the economic burden of pharmacotherapy.

## INTRODUCTION

In the contemporary Evidence-Based Medicine (EBM) era, randomised controlled trials (RCTs) guide recommendations, providing the gold standard for assessing the efficacy of pharmacological therapies or other interventions.^1^ However, in Rheumatology, important differences exist regarding the efficacy–effectiveness gap between RCTs and the real world, with most trials enrolling patients with better prognoses, thus overestimating the treatment effect.^2^ For instance, most of the trials examining new immunomodulatory drugs in Sjögren’s disease apply a similar study design, while enrolling patients who do not allow for generalisation of the findings (the majority with positive anti-SSA antibodies and an ESSDAI ≥5 or 6).^3^ Pharmaceutical-sponsored RCTs do not seem to fit well with the urgent need to improve clinical decisions in a manner that affects both policy, and individual patients.^4^

Furthermore, many patients are non-responsive to the delivered treatments.^5^ Secondary non-response consists of an additional issue: although an initial response is observed, the effectiveness of the therapy is diminished over time^6,7^ as a possible result of treatment persistence. These cases are labelled as “difficult to treat” and the patients are often suspected of being non-adherent to the therapeutic regime, due to personal reasons.^8^ Research and clinical experience indicate that one size does not fit all patients for many rheumatic diseases. For example, in systemic lupus erythematosus (SLE), RCTs reveal that an agent may significantly improve an endpoint in one trial, but fail to do so in other studies.^9,10^ Additionally, although the same pathway is broadly targeted by many trials, success and failure rates are similar.^11^ As for the efficacy of the active intervention against the placebo arms, this has been calculated to range between 10 to 20% even among positive trials; thus, when the economic costs associated with therapy are also considered, the number of patients with SLE benefiting from effective and patient-acceptable therapies is diminished.^11^

What we must consider is the individual nature of each patient’s case in this equation. The application of standardised treatments using clinical algorithms can lead to unresponsive patients who may receive ineffective therapy simply because it is the standard of care. Besides non-responsiveness, when assigning a patient to a specific therapeutic regimen, it’s essential to consider whether an alternative treatment might be more effective, as well as potential side effects, patient preferences, disease progression, or overall quality of life. N-of-1 trials allow each patient to serve as his/her control, testing different treatments in search of the most appropriate one. Thus, *n*-of-1 trials use the opportunity to individualise medicine and health care, addressing each patient as a unique case, allowing one to experiment (under control) in search of the best available treatment. According to Pitzalis,^12^ the diverse cellular and molecular features of rheumatic diseases indicate distinct clinical and treatment-response phenotypes, as revealed by omics studies. Thus, the conduction of *n*-of-1 trials is helping us move towards patient-centric precision medicine.^12^

### Unfolding the n-of-1 trial design

**Figure 1.**
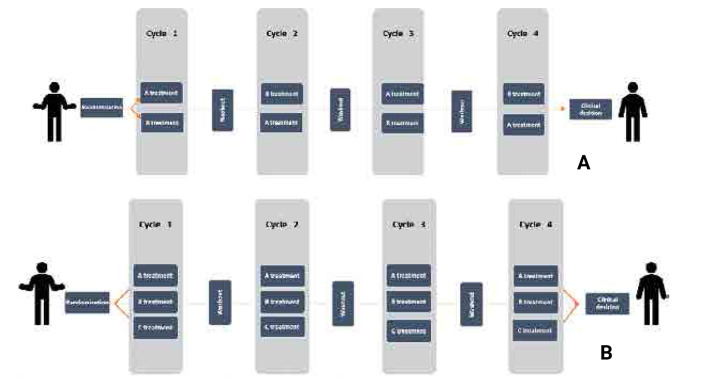
N-of-1 trials with two (**A**) and three (**B**) interventions.

By employing each patient as their control, these trials have the potential to minimise differences between subjects and significantly decrease overall error, consequently enhancing the statistical power of the trial. The Sicily Statement on Evidence-Based Practice (EBP) states that it requires healthcare decisions to be based on the best available, current, valid, and relevant evidence, while the care recipient should make these decisions under the care provider’s guidance.^13^ N-of-1 trials are single-subject, randomised, crossover (double-blinded wherever possible) clinical trials where a single patient serves as a control for the whole duration of treatment and constitutes a potential way for precise and person-centred EBP.^14^ The idea is that the patient is the only person suitable to act as a control for themselves since everyone has a unique genetic background, lifestyle, and environmental exposure.^15^ N-of-1 trials provide robust evidence, since they contain the same elements as RCTs, namely randomisation, outcome definition and quantification, patient and physician blinding and strict eligibility criteria.^16^ The patient undergoes each examined intervention, and repetition of the intervention phases provides statistical power. Repetitions of the so-called “AB paradigm” defend the trial against random error, while the design can include more than two interventions (placebo or active) if necessary^17^ (**Figure 1**).

Limitations of the design include the fact that after the first few cycles, each next cycle contributes little to the precision of the trial.^18^ In parallel, the consecutive application of different interventions might influence the outcomes due to potential carry-over effects. For this, asides from incorporating appropriate washout periods, researchers can employ randomisation in longer blocks and evaluate the gathered data after each period to mitigate these issues.^16^

Several prerequisites must align for applying *n*-of-1 trials, including the existence of a chronic condition, uncertainty regarding the treatment, variability in individual responses, incorporation of short and safe washout periods and well-defined outcomes.^19^ Regarding ethical aspects, the cornerstones remain the same as in RCTs: informed consent, confidentiality, and freedom to withdraw without consequences.^20^ Patient active involvement is highly encouraged, as *n*-of-1 trials increase patient awareness and understanding of the disease, allowing them to bear greater responsibility concerning therapeutic decisions, which leads to better health outcomes.^21^

The crucial difference between an *n*-of-1 trial and an RCT is that the former’s results are not generalisable but apply only to the specific patient. Differences between RCTs and *n*-of-1 trials are presented in **Table 1**. The efficacy of an intervention is determined by various factors, although it may not always be directly applicable to an individual patient. Several statistical methods have been proposed to combine the results of individual n-of-1 trials, including random-effects models and hierarchical Bayesian analysis, in a similar way that meta-analyses combine the results of RCTs.^22^

**Table 1.** Key differences between n-of-1 trials and randomised controlled trials.

**Aspect**	**N-of-1 trial**	**Randomised control trial**
**Study design**	Single participant, repeated measurements over time	Multiple participants randomly assigned to groups
**Purpose**	To determine the most suitable treatment for an individual	To assess the efficacy and safety of an intervention
**Randomisation**	Can involve randomisation	Randomised assignment of participants to intervention or control groups
**Sample size**	One participant	Larger size
**Control group**	The participant is their own control	Separate control group for comparison to the intervention
**Intervention type**	Tailored to participant	Standard intervention
**Generalisation**	Limited	Findings can be generalised to a broader population
**Statistical power**	Low	High
**Cost and time**	Lower cost, shorter duration	Higher cost and duration

### When to use the n-of-1 design in rheumatology

According to the Food and Drug Administration (FDA),^23^ 1 in 25 patients are non-responders to their medication due to genetic factors. These genetic factors may render therapies useless for that specific patient or prevent using them due to extreme adverse events resulting from genotype.^24^ To address this issue clinically, the FDA draft guidance^23^ provided a basic framework for clinicians, researchers, and the industry on the conduction of *n*-of-1 trials. It was also suggested that patients and their families collaborate in these small trials to identify the ideal therapy for each patient, addressing inherent heterogeneity in treatment response. Nearly 25% of patients with rheumatic disease cannot be definitively diagnosed, with the majority remaining undiagnosed for 5–10 years of follow-up.^25,26^ N-of-1 trials can serve this population, as different treatments can be tested alternately, aiming to identify the most efficient one. In 2007, Pincus^27^ suggested that the most logical therapeutic regime for any patient with early undifferentiated arthritis is conducting an immediate *n*-of-1 trial. This included testing weekly low-dose methotrexate (MTX) against 5 mg prednisone or less, for those where medical history and physical examination suggest inflammatory arthritis.^27^ The proposed approach aimed to treat some patients who may have had fibromyalgia syndrome (FMS) or post-infectious polyarthritis,^27^ avoiding possible overtreatment.

With rheumatologists being urged to treat, patients await treatment and symptom improvement.^28^ As many autoantibodies have low specificity for a definite diagnosis, especially during the early stages of the disease, overdiagnosis and overtreatment may well occur.^29^ Several researchers argue that overdiagnosis and over-treatment consists of pivotal problems in Rheumatology,^28,30^ issues hardly discussed but indeed present. They can lead to unnecessary medical use, patient anxiety and economic cost, and even severe toxicity, as experienced in the USA, where more than 150,000 deaths from painkillers occurred as the residue of opioid-induced hyperalgesia.^31^ In parallel, they can also worsen patient symptoms, health status, and condition, especially in those over-diagnosed for the means of justifying a drug test.^28^ Unarguably, preventive medicine is an attractive proposition in Rheumatology.^32^ However, the evidence has shown that although early treatment may well halt autoimmunity and prevent disease progression in some patients, this delay is inevitable in others.^32^ According to Berthelot,^28^ RCTs’ methodology, aims, and results cannot be transposed at the individual patient level without caution. In this manner, *n*-of-1 trials can assist in identifying the patients most likely to benefit from specific treatments, compared with those for whom treatment will be unnecessary and harm is expected to occur.^32^

The design can also target the reduction of healthcare costs and obtain cost offsets, for example, by determining the individual patients who do not benefit further from non-steroidal anti-inflammatory drugs (NSAIDs) use.^33,34^ In parallel, *n*-of-1 trials can serve as an economically viable option to improve access to selected high-cost medications.^35^ Identifying complementary and alternative medicines (CAM) that are effective for each patient and can be added to the usual care can also be determined using this research design. ^36,37^

An additional advantage is that the *n*-of-1 design can determine the best treatment in some rare autoimmune rheumatic diseases, providing insight into individual efficacy and safety.^38^ In a similar manner, employing this design may well benefit younger populations, where achieving an adequate sample size is difficult for the conduction of traditional trials.^39^ Last but not least, given that most rheumatic diseases do not follow a predictable progression, employing this methodology provides the chance to observe the patient’s response to various therapeutic treatments and thoroughly characterise the patient’s unique phenotype. N-of-1 trials can be combined with Bayesian hierarchical models to provide population comparative treatment effect estimates.^18^

### The niche of n-of-1 trials for biological agents

Introducing biological agents offered additional areas for conducting *n*-of-1 trials in Rheumatology, using less costly approaches and less restrictive management regimes compared with mandatory stepped care.^40^ Twenty-five percent of patients switch agents within the first two years of initiating a first biological agent, with many reaching their third or fourth biological medication in search of symptom relief.^41^ Additionally, many patients chronically control their disease poorly despite the prescription of 1–5 biological agents.^42^ The use of immune-suppressing therapies, which are ineffective for specific individuals, is associated with a plethora of risks for the development of adverse effects, reduced quality of life, and physical function due to uncontrolled disease.^43^ Although the *n*-of-1 design seems ideal for testing the individual efficacy of biologicals, no such study has been implemented to date. In a hypothetical trial, offering patients with rheumatoid arthritis (RA) the opportunity to participate in an *n*-of-1 trial comparing MTX with etanercept (ETA) can reduce total health care and medication costs while securing the equipoise principle of no harm.^40^ Although offering an *n*-of-1 trial was initially more expensive than stepped care by 15%, the final economic costs were 47% cheaper than the open ETA access.^40^

In real-world studies,^44^ even in cases of difficult-to-treat RA, treatment retention is low in modern drugs like Janus Kinase (JAK) inhibitors, with 49% of the patients discontinuing treatment at 12 months due to lack of effectiveness, adverse events, or other reasons. Thus, patients may often need more sophisticated treatment options when considering individual efficacy and weighing the benefits and harms of specific treatments.

### Studies incorporating the n-of-1 trial design

#### Search strategy

For the purpose of this review, we searched PubMed and clinicaltrials.gov for *n*-of-1 trials or series of *n*-of-1 trials conducted on patients with rheumatic diseases until August 2024. Two independent reviewers used a combination of MeSH terms and keywords including (juvenile arthritis), (juvenile idiopathic arthritis), (rheumatoid arthritis), (psoriasic arthritis), (vasculitis), (Raynaud*), (osteoarthritis), (lupus), (systematic lupus erythematosus), (Behcet*), (fibromyalgia), (Sjögren*), (spondylarthritis), (myositis), (dermatomyositis), (polymyositis), (Wegener*), (Buerger*), (Cogan), (Henoch-Schönlein), (Churg-Strauss), (polyangiitis), (antiphospholipid antibody syndrome), (antiphospholipid syndrome), (gout), (uric arthritis), (Ankylosing Spondylitis), (systemic sclerosis), (scleroderma), (Polymyalgia Rheumatica), (Giant Cell Arteritis), (rheumatic disease*), (*n*-of-1), etc. We selected n-of-1 trials or series conducted on patients with any rheumatic disease diagnosis.

#### N-of-1 trials for Juvenile Idiopathic Arthritis (JIA)

Huber^45^ was the first to conduct a series of *n*-of-1 trials in children and adolescents with juvenile idiopathic arthritis (JIA) (**Table 2**). The aim was to assess the ability of this design to estimate the population effect of delivered therapeutic interventions and determine whether low-dose amitriptyline (AMP) in particular, may reduce pain in a significant manner in children with active polyarticular course JIA, between 10–18 years old. The results revealed a very low probability that AMP reduced pain, since children also reported experiencing significant pain reduction when on placebo.^45^ Nonetheless, the application of *n*-of-1, analysed using Bayesian statistics, was deemed efficient in estimating the population effect of an intervention.^45^

**Table 2.** N-of-1 trials conducted in patients with FMS, JIA, RP and AAV.

**First author**	**Jaeschke^59^**	**Zucker^60^**	**Huber^45^**	**Lee^46^**	**Roustit^49,50^**	**Scott^47^**
**Design**	Series of *n*-of-1	Series of n-of-1	Series of *n*-of-1	Series of *n*-of-1	Series of *n*-of-1	Series of *n*-of-1
**Diagnosis**	FMS	FMS	JIA	JIA	RP	AAV
**Origin**	Canada	USA	Canada	U.K.	France	Ireland, UK, The Netherlands
**Registry**	NR	NCT00000428	NR	NR	NCT02050360	
**Concealment**	Double blind	Double blind	Double blind	Open label	Double blind	Single blind (investigators blinded to UV data)
**Multicentre**	No	√	√	√	No	√
**Randomisation**		A central pharmacy prepared blinded, random-ordered treatment kits and block-randomised first treatment sets	PC-generated random number list, placed in sealed, numbered envelope	Using a random number list, generated before recruitment (PI), with block randomisation	Using a block size of 6, so the same sequence could not be repeated for the same person	N/A
**Participants**	N=23 patients with FMS	N=58 patients with FMS	N=14 children and adolescents with JIA	N=14 children and young people with JIA (5–16 years old) and their parents	N=38 patients with primary or secondary RP	N=439 patients with definite AAV diagnosis (clinical features of GPA, MPA or EGPA, with either positive anti-MPO or PR3 serology and/or histopathology)
**Recruitment**	Single Rheumatology practice	Referral centre and community practices, Boston	Hospital for Sick Children, Toronto, and The IWK Health Centre, Halifax	CAPS, Manchester	Dept of Vascular medicine,Grenoble Alpes University Hospital	Irish RKD
**Aim**	- Assess time of onset and cessation of action of AMT for FMS - Assess range of AMT doses that are useful for FMS - Assess usefulness of n-of-1 to reach a clinical decision	Compare FMS therapies and assess feasibility and outcomes of this approach for effectiveness research	- Assess if low-dose AMT in children aged 10–18 yrs with active poly-articular JIA reduces pain in a clinically significant manner - Assess feasibility of *n*-of-1 trials analysed using Bayesian statistics, in estimating the population effect of an intervention	Explore the use of 4 different time sampling strategies: once-a-day, BID, once-a-week, and as-and-when children and young people had pain	- Assess the efficacy and safety of on-demand SDF in RP - Assess the placebo response in RP, and evaluate the level of RTM contribution to this response, using trial and individual-level data	Assess the association between measures of ambient UV radiation, UVB-predicted vit D status (CW-D-UVB) and AAV relapse
**Total intervention phases**	3 (ABA)	6 (ABBABA)	6	8 (ABCDBADC)	At least 6 (2 blocks)	NR
**Intervention (Phase A)**	AMP (5–50 mg at night)	AMP (placebo in the morning and 25 mg AMP at night)	AMP (25 mg by mouth 1–2h before bedtime)	Once-a-day pain reporting using the MPT	SDF (40 mg/dose at a maximum of 2 doses/day)	Relapse (signs/symptoms of active vasculitis [BVAS], supporting laboratory or histopathological evidence, escalation in immunosuppression, immuno-suppression response)
**Intervention (Phase B)**	Placebo	AMP+FL (20 mg FL in the morning + 25 mg AMP at night)	Placebo	Twice-a-day pain reporting using the MPT	SDF (80 mg/dose at a maximum of 2 doses/day)	Remission (absence of signs, symptoms and laboratory evidence of vasculitis activity)
**Intervention (Phase C)**	-	-	-	Once-a-week pain reporting using the MPT	Placebo	-
**Intervention (Phase D)**	-	-	-	As-and-when pain reporting using the MPT	-	-
**Duration of each Phase**	2 weeks (at first it was 4 weeks)	6 weeks	2 weeks	1 week	1 week	Evaluated based on patient signs and symptoms
**Washout duration**	None	None	1 week	1 day		
**Outcomes**	7-question symptom questionnaire, TPE	FIQ, specifically gauging global well-being, pain, sleep, fatigue (VAS) and feeling refreshed upon awakening, TPE, AEs, patient perceptions	Pain (VAS), physical function (CHAQ), sleep quality, fatigue (FSS), morning stiffness	Pain Interference (PROMIS PPPISSF)	Condition score (RCS), frequency and daily duration of RP attacks, skin blood flow in response to cooling	Winter vitD-UVB, annual vitD-UVB, CW-D-UVB
**AEs**	NR	AEs were reported (N=38 patients) leading to 8 withdrawals. The most frequent AEs involved sedation, headache, dryness, and GI-related symptoms	No patient experienced serious AEs and none withdrew. All patients complained of at least one AE at some point including nausea, sedation, blurred vision, dry mouth, diarrhoea, and sore throat, without difference between arms	Two participants experienced technical difficulties during the study, during which time, MPT data was not saved to iPads	One patient discontinued due to symptomatic hypotension. Other reported AEs included dyspnoea, headache, flush, GI disorders, chest pain, fatigue, spontaneous erection, finger cyanosis, insomnia, epistaxis, dizziness, visual disturbances	NR
**Results**	Patients expressed confidence in the treatment decision (74%). For 35% of patients, results AMP, which would other-wise have led to cessation of been continued indefinitely. The drug benefit was evident within the first 2 treatment weeks.	The pooled results revealed a better outcome score in those on combination therapy. These population results are similar to published Neither practice type, nor patient characteristics were outcomes from a more traditional crossover trial. associated with the observed treatment-effect variation. Most patients, irrespective of selected treatment, felt their trials were helpful.	A very low probability was revealed that AMP reduced pain in the sample, as the mean treatment effect for pain greater pain reduction when on placebo. The was 0.67, indicating that children experienced a results of n-of-1 trials, analysed using Bayesian statistics were efficient in estimating the population effect of an intervention.	Adherence to pain reporting was higher in less intense time sampling strategies (63% in once-a-week) compared with more intense time differences were noted in pain interference scores sampling strategies (37·8% in BID). No statistically between sampling strategies. Qualitative interviews indicated preference for once-a-day (43%) and as-and-when pain reporting (43%).	The probability that the efficacy of SDF 40 mg is superior to placebo on the frequency of RP was 93%, and 90·6% for the RCS; and 91·5% and 62·6%, SDF induced more AEs than placebo. SDF had a dose respectively, for SDF 80 mg. -dependent effect on skin blood flow during cooling. A large, significant placebo response was noted from individual and trial data for RCS and RP frequency.	Latitude was related to AAV relapse risk. An inverse link was noted between relapse risk and average winter and annual vitD-UVB, with a larger effect size in observed between relapse risk and both average and MPA. An inverse link was preceding winter CW-D-UVB. Other relapse risk factors included GPA/EGPA subtype, younger age, being off immunosuppression therapy or on GC monotherapy.

AAV: ANCA-associated vasculitis; AEs: adverse events; AMP: amitriptyline; ANCA: Anti-neutrophil cytoplasm antibody; anti-MPO: anti-myeloperoxidase; BID: twice-a-day; BVAS: Birmingham Vasculitis Activity Score; CAPS: the Childhood Arthritis Prospective Study; CDSA: Comprehensive Digestive Stool Analysis; CHAQ: Childhood Health Assessment Questionnaire; CW-D-UVB: cumulative-weighted UVB dose; EGPA: Eosinophilic granulomatosis with polyangiitis; FIQ: Fibromyalgia Impact Questionnaire; FL: Fluoxetine; FMS: fibromyalgia syndrome; FSS: Fatigue Severity Scale; GC: glucocorticoid; GI: gastrointestinal; GPA: granulomatosis with polyangiitis; JIA: juvenile idiopathic arthritis; MPA: microscopic polyangiitis; MPT: My Pain Tracker; N/A: not applicable; NR: not reported; NSAIDs: non-steroidal anti-inflammatory drugs; PI: principal investigator; PPPISSF: Parent Proxy Paediatric Pain Interference Scale-Short Form; PR3: proteinase 3; PROMIS: Patient-Reported Outcomes Measurement Information System; RCS: Raynaud Condition Score; RKD: Rare Kidney Disease; RP: Raynaud’s Phenomenon; RTM: Regression towards the mean; SDF: Sildenafil; TPE: tender point examination; UV: Ultraviolet; UVB: Ultraviolet B; VAS: visual analogue scale; vitD: vitamin D; vitD-UVB: Daily UVB data at wavelengths specific for vitamin D production.

Additional *n*-of-1 trials were conducted in children and adolescents with JIA^46^ (**Table 2**), aiming to explore four distinct time sampling strategies for reporting pain using a mobile health (mHealth) app, My Pain Tracker (MPT). The four strategies involved reporting pain in MPT once or twice a day, once a week, and as-and-when. Creating a pain-reporting routine was one of the most important factors for successful outcomes. Patient reporting preferences are key to accommodating disease self-management and crucial when data capture quality is sought.

#### N-of-1 trials for ANCA-associated vasculitis (AAV)

Scott and associates^47^ applied the *n*-of-1 design to evaluate the association between ambient ultraviolet B (UVB) dose and antineutrophil cytoplasmic autoantibody (ANCA) -associated vasculitis relapse (**Table 2**). Each patient served as his/her own control when on relapse (phase a) or remission (phase b) and residential latitude, daily UVB data at wavelengths specific for vitamin D production (vitD-UVB) and cumulative-weighted UVB dose (CW-D-UVB) were evaluated. In this non-traditional *n*-of-1 trial, intervention phases involved the relapse and remission phases of each patient. The results revealed that residential latitude was positively correlated and average vitD-UVB was negatively correlated with relapse risk, with a stronger effect among winter-only measurements. These associations were not restricted to granulomatous phenotypes but the study provided useful information regarding the effect of vitamin D and sup exposure in ANCA-associated vasculitis.

#### N-of-1 trials for Raynaud’s phenomenon (RP)

Raynaud’s phenomenon (RP) is commonly encountered in rheumatology as the epiphenomenon of systemic sclerosis (SSc).^48^ Using the *n*-of-1 design, Roustit and colleagues^49,50^ assessed the effect of phosphodiesterase-5 (PDE5) inhibitors, and in particular sildenafil (SDF), in men and women with primary or secondary RP (**Table 2**). The results showed that the probability that SDF was more efficient than placebo was greater than 90% for all outcomes.^49^ In parallel, the substantial heterogeneity observed in SDF efficacy among patients indicated the need for individualising RP treatment, highlighting the value of *n*-of-1 trials. Interestingly, a large and significant placebo response was observed,^50^ adding further value to the use of *n*-of-1 trials in these patients.

#### N-of-1 trials for osteoarthritis (OA)

Chronic pain is a prevalent, clinically vexatious condition that places a high economic burden on societies and the healthcare system. For pain management, clinicians typically apply the trial-and-error approach, although repeated crossover trials of single patients in the form of *n*-of-1 trials may provide improved therapeutic precision.^51^

In 1994, the first *n*-of-1 trial in patients with chronic pain was conducted.^52^ To date, seven publications (Table 3) report *n*-of-1 trials conducted on patients with osteo-arthritis (OA),^33,52–57^ six of which consist of a series of trials^33,52,54–57^ (one as part of a parallel trial),^33^ and the latter being a single patient case.^53^ Most researchers aimed to reduce unnecessarily prolonged treatment with NSAIDs and pain intensity in OA by investigating individual patient efficacy of NSAIDs against paracetamol (PCT) or placebo,^33^ in an effort to facilitate deprescribing. One trial series compared the application of an NSAIDs topical gel against capsaicin cream,^54^ while Brooks^57^ assessed the feasibility of conducting *n*-of-1 trials in patients with OA.^57^ The final trial investigated complementary medicines by delivering probiotics compared to placebo.^53^ Pope^33^ calculated the economic burden associated with NSAID use among patients with OA, revealing double costs compared with conventional treatments. According to a recent systematic review, *n*-of-1 trials have proved efficient in solving refractory cases in OA and chronic musculoskeletal and neuropathic pain.^58^ As a result, various trials are currently being conducted in search of the most appropriate individualised treatment for chronic pain, including patients with OA.^51^

**Table 3.** N-of-1 trials conducted in patients with OA.

**First author**	**Brooks^57^**	**March^52^**	**Persson^54^**	**Pope^33^**	**Taye^53^**	**Wegman^55^**	**Yelland^56^**
**Design**	Series of *n*-of-1	Series of *n*-of-1	Series of *n*-of-1	Series of *n*-of-1, as part of parallel trial	*n*-of-1 trial	Series of *n*-of-1	Series of *n*-of-1
**Diagnosis**	Knee OA	OA	OA	OA	OA	OA	OA
**Origin**	U.K.	Australia	U.K.	Canada	Australia	The Netherlands	Australia
**Registry**	NCT00371696	NR	NCT03146689	NR	NR	NR	NR
**Publication**	Trials., 2007	BMJ, 1994	Rheumatology (Oxford)., 2021	J Rheumatol., 2004	Complement Ther Med., 2020	Ann Rheum Dis., 2003	Rheumatology, 2007
**Concealment**	Triple blind	Triple blind	Open label	Double blind	Double (?) blind	Triple blind (statistician aware)	Double blind
**Multicentre**	√	√	NR	NR	-	√	√
**Randomisation**	Determined by an independent researcher using PC-generated random numbers	NR	Using a web-based program by a researcher not involved in the study	“in random order”, with the code broken once preference was observed	By independent researcher using Excel to generate a random sequence	Prepared in advance by the hospital pharmacist	Using a PC-generated schedule
**Participants**	N=9 with confirmed OA of the knee	N=25 patients with OA	N=22 patients with chronic knee pain and radiographic knee OA	N=24 patients with OA, uncertain that NSAIDs were helpful	N=1 67- year-old woman with OA in her lower back and right ankle	N=22 patients with OA, receiving NSAIDs regularly	N=59 patients with OA and severe pain, warranting inspection for long-term CLC use, with doubt regarding efficacy
**Recruitment**	Clinics, North Bristol Health Care Trust	GPs, metropolitan Sydney	Nottingham KPIC cohort study	NR	Naturopath	25 GPs in Amsterdam	Invitations by post
**Aim**	Explore feasibility and patients' perspectives of being involved in their own n-of-1 trial	Evaluate the efficacy of PCT and an NSAIDs for symptom relief in OA	Determine individual responses to IBU gel/capsaicin cream for painful, radiographic knee OA	Determine patients who truly no longer need NSAIDs, in order to reduce treatment costs	Determine the effectiveness of probiotics on OA pain in one patient	Determine if PCT is as effective as NSAIDs for pain and OA-related disability among people using NSAIDs regularly	Assess the efficacy of SR PCT with CLC in individual patients
**Total intervention phases**	6	6 (ABBABA)	6 (ABBAAB)	6 (ABBABA)	6 (ABBABA)	10 (ABBABAABBA)	6 (ABBABA)
**Intervention (Phase A)**	500 mg tablets of PCT thrice/day	PCT (1 g BID)	5% IBU gel	DIC (one pill BID)	2 caps/day probiotics (*Lactobacillus rhamnosus, Saccharomyces cerevisiae boulardii and Bifidobacterium animalis ssp lactis*)	NSAID (same dosage, as before the study)	CLC (200 mg/day, or 200 mg BID for those already using this dose)
**Intervention (Phase B)**	One 50 mg tablet of DIC thrice a day	DIC (50 mg BID)	0.025% capsaicin cream	Placebo (one pill BID)	Placebo	PCT (dosage adjusted to that of the NSAID)	SR PCT (1330 mg) thrice/day
**Intervention (Phase C)**	Placebo thrice/day	-	-	-	-	-	-
**Intervention (Phase D)**	-	-	-	-	-	-	-
**Duration of each Phase**	2 weeks	2 weeks	4 weeks	2 weeks	3 weeks	2 weeks	2 weeks
**Washout duration**	None	NR	Individualised (max 4 weeks)	None	2 weeks	1 week	NR
**Outcomes**	Interviews exploring reasons for participation and experiences, as well as understanding the trial design	Pain and stiffness, function, and side effects	Pain (NRS)	Health assessment, WOMAC	Pain (VAS), patient preference, health assessment, PSFS, CDSA, rescue medication use	Individual main complaints and pain intensity	Pain, stiffness, functional limitation, preferred medication, AEs, Δ in drug use
**Drop-outs**		n=5 withdrew but had made a therapeutic decision, and n=5 dropped out very early	*n*=5 withdrew before completion (3 for erythema and skin irritation following IBU gel, 1 withdrew consent, and 1 died during 3^rd^ washout )	*n*=3 drop-outs	-	*n*=9 were excluded, *n*=6 did not complete the study	N=18 completed 1 or 2 cycles (CLC AEs, severe pain, 5 due to the TGA directive; large amount of tablets, concern for AEs, failure to complete diaries, and impending admission for surgery)
**Results**	Patients were keen to participate, believing that the trial may lead to personal gains such as improved symptom control and quality of life. However, recruitment to the pharmacological comparison was more difficult since this could also entail risk. All patients were eager to complete the trial, even when difficulties were encountered.	Several (8/20) patients showed no difference, symptoms being adequately controlled by PCT; five preferred the NSAID; two controlled symptoms after their initial 2 weeks of the NSAID which continued at subsequent treatment changes; in five the NSAID may have been better but neither agent gave satisfactory control. After 3 mo, 9/20 patients on PCT had adequate symptom control.	Irrespective of equal efficacy observed, 59% of patients displayed a greater response to one treatment over the other. Thus, those who fail to benefit from one type of topical treatment should be offered another, which may be more effective.	Nearly half (11/21) patients preferred DIC. None of the n-of-1 patients preferred placebo and 11 had no preference. At 6 mo, 17/21 were on NSAIDs. NSAIDs were deemed as effective in 81% of n-of-1 subjects and 79% of conventionally treated patients, even though subjects were initially uncertain if NSAID were helpful.	The reduction in pain scores associated with the probiotic was small, but clinically significant for this patient. A holistic view of the patient focusing on digestive integrity and function may be crucial for clinical applications of interventions such as probiotics.	For 5/7 patients completing the study, little/no difference was noted between PCT and NSAIDs; for one patient the results favoured NSAID, and for one no association bet-ween outcome and drug was noted. Six patients were advised to change to PCT, the rest continued on NSAIDs. Three mo after the study, 4/6 patients on PCT Rx were on NSAIDs for practical reasons, or perceived lack of efficacy.	On average, CLC had better scores than SR-PCT [pain, stiffness and functional limitation], 33/41 patients (80%) failed to spot differences between SR-PCT and CLC in symptom relief. Of the 8 patients who noted differences, 7 had better relief with CLC and 1 with SR-PCT. In 25/41 (61%) patients, subsequent management was consistent with trial results.

AEs: adverse events; AMP: Amitriptyline; BID: twice a day; CDSA: Comprehensive Digestive Stool Analysis; CLC: Celecoxib; DIC: Diclofenac; FL: Fluoxetine; GPs: General Practitioners; IBU: Ibuprofen; KPIC: Knee Pain and Health in the Community; Mo: Months; NRS: numeric rating scale; NSAIDs: non-steroidal anti-inflammatory drugs; OA: osteoarthritis; PC: personal computer; PCT: Paracetamol; PSFS: Patient Specific Functional Scale; Rx: medication prescription; SR: sustained-release; TGA, Therapeutic Goods Administration; VAS: visual analogue scale; WOMAC: Western Ontario and McMaster Universities Osteoarthritis Index; Δ: difference.

#### N-of-1 trials for Fibromyalgia syndrome

Regarding fibromyalgia syndrome (FMS) two *n*-of-1 trials have been conducted to date (**Table 2**). Jaeschke^59^ assessed the time of onset and termination of action of AMP for FMS and the range of AMP doses that were useful for treating FMS. The main goal however, was to evaluate the usefulness of the *n*-of-1 design in reaching a clinical decision regarding FMS therapy. At the end, physicians had confidence in the treatment decisions of most of the included patients (74%). For 35% of patients, AMP was not different from the placebo and was hence discontinued; otherwise, it would have been continued indefinitely. The drug benefit was evident within the first two weeks of treatment, indicating that the *n*-of-1 design can be useful in FMS.

Zucker^60^ compared FMS therapies (AMP versus combined AMP and fluoxetine), assessing the feasibility and outcomes of the *n*-of-1 approach. Most patients reported feeling that their *n*-of-1 trials were useful in choosing the proper treatment. On the other hand, the pooled results revealed a better outcome score in combination therapy compared to AMP alone. Thus, implementing the combined *n*-of-1 methodology is feasible in Rheumatology practice and may improve FMS symptom management.

Overall, patients with FMS are dissatisfied with existing services^61,62^ and feel frustrated due to the lack of efficacy of conventional treatments. Paired with the variable response to treatment solutions, these factors could justify the introduction of *n*-of-1 trials in FMS management.

### In the pipeline

Currently, many scientific and research institutions are turning the spotlight on *n*-of-1 trials for rheumatological everyday practice. The Tufts Clinical and Translational Science Institute (CTSI) has initiated a series of n-of-1 trials for patients with RA^63^ (**Table 4**). As treatments are often selected by physicians based on the clinical practice guidelines’ therapeutic algorithms, considering patients’ experiences and adverse events may change what is deemed “ideal treatment”. Furthermore, the Tufts Trial Innovation Network (TIN) Design Lab has also initiated the design and implementation of a series of *n*-of-1 trials for childhood Sjögren’s disease (cSD), aiming to assess the efficacy of potential interventions on the mucosal/glandular manifestations of cSD.^64^ This approach aims to overcome the limitations of traditional parallel-group trial designs, including ensuring the power of the trial, given the rarity of cSD. Similarly, the University of Texas Health Science Centre at Houston (UTHealth Houston) recruits patients with axial spondyloarthritis (AxSpa) for *n*-of-1 trials assessing the efficacy of individualised anti-inflammatory medications.^65^ Results of the AxSpa trials are awaited, whereas the RA trials have yet to start recruiting patients. In parallel, the *n*-of-1 trial design has also been suggested for evaluating candidate treatments in SSc,^66^ SD,^64^ idiopathic inflammatory myopathies^67^ and inclusion body myositis^68,69^ at the individual patient level. Finally, OA treatment is currently being examined using the *n*-of-1 format, comparing naproxen to placebo.

**Table 4.** Ongoing n-of-1 trials conducted in patients with rheumatic diseases.

**CTI**	**Site**	**Patients**	**Interventions**	**Outcomes**
**NCT06016517^†^**	Tufts Medical Centre, USA	N=18, patients with RA	- ADA (40 mg SC every 2 weeks) with oral placebo once daily - SRL (200 mg SC every 2 weeks) with oral placebo once daily - UPA (15 mg) orally once daily with SC placebo injection every 2 weeks	Δ in DAS28, ESR, CRP, TSJ, SF-12, RAPID3, TBQ, PROMIS PF10a
**NCT04115098^‡^**	University of Texas Health Science Centre, USA	N=42, patients with axSpa	- NPX (500 mg) tabs BID - MLX (7·5 mg ) tabs BID and - CLC (200 mg) caps BID	Δ in ASDAS, BASFI, QoL, PROMIS-29, BASDAI, BASMI, Pain (VAS)
**NCT05430230**	Northwestern University, USA	People with painful knee OA	- NPX (500 mg) - Placebo	Δ in KOOS, pain (PDQ), PANAS, QST, disability (ODI)

ADA: Adalimumab; ASDAS: Ankylosing Spondylitis Disease Activity Score; axSpa: axial spondyloarthritis; BASDAI: Bath Ankylosing Spondylitis Disease Activity Index; BASFI: Bath Ankylosing Spondylitis Functional Index; BASMI: Bath Ankylosing Spondylitis Metrology Index; BID: twice-a-day; caps: capsule; CLC: Celecoxib; CRP: C-reactive protein; CTI: clinical trial identification; DAS28: Disease Activity Score-28; ESR: erythrocyte sedimentation rate; KOOS: Knee Injury and Osteoarthritis Outcome Score; MLX: Meloxicam; NPX: Naproxen; ODI: Oswestry Disability Index; PANAS: Positive and Negative Affect Schedule; PDQ: Pain Detect Questionnaire; PF10a: Physical Function Short Form 10a; PROMIS: Patient-Reported Measure of Physical Function; QoL: quality of life; QST: Quantitative Sensory Testing; RA: rheumatoid arthritis; RAPID3: Routine Assessment of Patient Index Data 3; SC: subcutaneous; SF-12: Short-Form 12- item Health Survey; SRL: Sarilumab; tab: tablet; TBQ: Treatment Burden Questionnaire; TSJ: tender and swollen joints; VAS: visual analogue scale; UPA: Upadacitinib; Δ, difference.

† Not yet recruiting;

‡ Complete.

### A case vignette for rheumatologists

**Figure 2.**
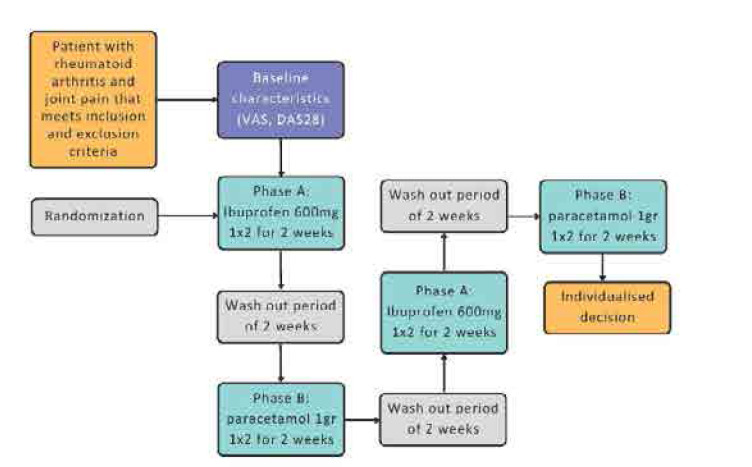
A case vignette of a hypothetical patient participating in an *n*-of-1 trial.

N-of-1 trials do not consist of a niche topic reserved for researcher only, but a valuable tool for everyday clinical practice. A rheumatologist often wants to determine which pain-relieving medication (for example NSAIDs or paracetamol) works better for a specific patient with joint pain (**Figure 2**). The patient has tried both medications in the past, but it is unclear which one is more effective. A *n*-of-1 trial would be ideal in this hypothetical scenario, as it will compare the effectiveness of the treatments for this specific patient, providing individualised recommendations.

As in every other trial, we start with the baseline assessment, evaluating the level of pain and daily functioning with specific metrics. For example, the pain can be measured using a Visual Analogue Scale (VAS) or disease specific metrics. Hypothetically, if the patient had a rheumatoid arthritis diagnosis, one could apply the DAS-28.^70^ In phase A, the patient is prescribed ibuprofen 600 mg b.i.d. for a set period of time, supposedly 2 weeks. During this time, pain intensity and any possible side effects –such as gastrointestinal events–are monitored and recorded. After phase A, a wash-out period of 1–2 weeks follows to ensure no residual effects. In Phase B the patient takes a standard dose of paracetamol (eg. 1 g b.i.d.) for two weeks, again tracking pain levels and any adverse events. Phases A and B can be repeated to increase reliability and provide more robust results.

At the end, patient’s responses to each regime are compared in terms of effectiveness, adverse events and patient satisfaction. In this manner, the rheumatologist can make a personalised recommendation tailored to the patient’s needs.

## SCIENTIFIC SOCIETIES AND CLINICAL PRACTICE GUIDELINES

N-of-1 trials can be used to improve prescription while, in parallel, aiding clinical decision-making.^71,72^ They can also evaluate the delivery of mHealth interventions^46,73,74^ or CAM17 and explore patient preferences and perceptions.^46,74^ N-of-1 trials are the epitome of personalised medicine, accounting for every aspect of EBM, including patient-reported outcomes and experience measures (PROMs/PREMs). Patient preferences and values are important when making health care decisions, and this also consists of an integral part of practicing EBM.^75^ Although they stand at the top of the evidence pyramid, none of the scientific societies associated with Rheumatology have recommended their use, suggesting horizontal treatment algorithms while overlooking that each patient is unique and has distinct drug responses and adverse events sensitivity.

## LIMITATIONS OF N-OF-1 TRIALS

Longer time and more efforts are needed from investigators when carrying out *n*-of-1 trials, which inevitably results in implementation difficulties.^58^ Furthermore, the *n*-of-1 design has limitations. For instance, washouts reduce carryover effects, but may also compromise patient safety.^76^ When tested drugs or interventions have a relatively long half-life –as several medications used in Rheumatology–, implementing the *n*-of-1 design becomes more challenging, as longer washout periods are required to reduce the carryover effect.^77^ Of note, even when washout periods are accounted for, the influence of a prior intervention may still exist on the endpoints of interest. At the moment we are unsure of the ideal washout duration for lifestyle interventions.^17^ Finally, it should be noted that when an effective intervention is stopped, the patient’s psyche is also affected, and this may additionally influence future interventions.^76^

## CONCLUSIONS

N-of-1 trials consist of the research design closest to the concept of individualised medicine,^68^ and for this, they are currently gaining residence in rheumatology. The design ensures scientific rigor, while generating clinically relevant treatment outcomes tailored to the patient involved. N-of-1 trials can be employed to facilitate clinical decisions, assess the efficacy of drugs, lifestyle interventions, and adjuvant treatments (as for pain or oral nutrient supplements), targeting the main symptoms of rheumatic diseases, as well as the frequent comorbidities (i.e., obesity, cachexia, Raynaud’s phenomenon, fatigue, etc.).^17,49,78,79^ The present review showed that introduction of the *n*-of-1 design in everyday clinical practice is feasible and useful and consists of the epitome of patient-centred medicine. The employment of *n*-of-1 trials in rheumatology can greatly benefiting patients and clinicians, while facilitating evidence-based deprescribing^80^ and reducing the economic burden of pharmacotherapy in rheumatic diseases.

## CONFLICT OF INTERESTS

The authors declare no competing interest.

## DATA SHARING

Not applicable.

## AUTHOR CONTRIBUTIONS

MGG and DPB conceived the study. MGG and AG wrote the manuscript and created the tables and figures. SGT and TS reviewed the literature. DPB and DGG supervised the work. All authors participated in manuscript corrections and take full responsibility for the integrity and accuracy of all aspects of the submitted work.

## FUNDING

No funding was secured for the present work.

## DISCLAIMER

No part of the manuscript has been submitted elsewhere. AI was not used for any part of the submitted work.
